# In vitro sepsis induces Nociceptin/Orphanin FQ receptor (NOP) expression in primary human vascular endothelial but not smooth muscle cells

**DOI:** 10.1371/journal.pone.0274080

**Published:** 2022-09-15

**Authors:** Mark F. Bird, Barbara Gallacher-Horley, John McDonald, David G. McVey, Fatin Al-Janabi, Remo Guerrini, Girolamo Calo, Shu Ye, Jonathan P. Thompson, David G. Lambert

**Affiliations:** 1 Department of Cardiovascular Sciences, Anaesthesia, Critical Care and Pain Management, University of Leicester, Leicester, United Kingdom; 2 Department of Chemical, Pharmaceutical and Agricultural Sciences, University of Ferrara, Ferrara, Italy; 3 Department of Pharmaceutical and Pharmacological Sciences, University of Padova, Padova, Italy; Gifu University School of Medicine Graduate School of Medicine: Gifu Daigaku Igakubu Daigakuin Igakukei Kenkyuka, JAPAN

## Abstract

Sepsis is a dysregulated host response to infection that can cause widespread effects on other organs including cardiovascular depression, hypotension and organ failure. The receptor for Nociceptin/Orphanin FQ (N/OFQ), NOP is expressed on immune cells and these cells can release the peptide. Exogenous N/OFQ can dilate blood vessels and this peptide is increased in animal and human sepsis. We hypothesise that NOP receptors are present on vascular endothelial cells and therefore provide the target for released N/OFQ to cause vasodilation and hence hypotension. Using human umbilical vein endothelial cells (HUVEC) and human vascular smooth muscle cells (HVSMC) freshly prepared from umbilical cords and up to passage 4, we assessed NOP mRNA expression by Polymerase Chain Reaction (PCR), NOP surface receptor expression using a fluorescent NOP selective probe (N/OFQ_ATTO594_) and NOP receptor function with N/OFQ stimulated ERK1/2 phosphorylation. As an *in vitro* sepsis mimic we variably incubated cells with 100ng/ml Lipopolysaccharide and Peptidoglycan G (LPS/PepG). HUVECs express NOP mRNA and this was reduced by ~80% (n = 49) after 24–48 hours treatment with LPS/PepG. Untreated cells do not express surface NOP receptors but when treated with LPS/PepG the reduced mRNA was translated into protein visualised by N/OFQ_ATTO594_ binding (n = 49). These NOP receptors in treated cells produced an N/OFQ (1μM) driven increase in ERK1/2 phosphorylation (n = 20). One (of 50) HUVEC lines expressed NOP mRNA and receptor protein in the absence of LPS/PepG treatment. In contrast, HVSMC expressed NOP mRNA and surface receptor protein (n = 10) independently of LPS/PepG treatment. These receptors were also coupled to ERK1/2 where N/OFQ (1μM) increased phosphorylation. Collectively these data show that an *in vitro* sepsis mimic (LPS/PepG) upregulates functional NOP expression in the vascular endothelium. Activation of these endothelial receptors as suggested from *in vivo* whole animal work may contribute to the hypotensive response seen in sepsis. Moreover, blockade of these receptors might be a useful adjunct in the treatment of sepsis.

## Introduction

Sepsis is a dysregulated host response to infection that can lead to life-threatening organ dysfunction and is responsible for up to 11 million deaths worldwide per year [1,2]. In certain cases, sepsis progresses to a more severe state, septic shock. Septic shock has been defined by the onset of hypotension (systolic BP 65mm Hg) with a serum lactate concentration of ≥2 mmol/L after fluid resuscitation [3]. Septic shock has a mortality rate of approximately 38% [4]. As with sepsis, the factors involved in the development of septic shock remain unclear.

The NOP/N-OFQ system has a number of effects on the cardiovascular system and may be a potential contributory mediator in sepsis. The NOP receptor is a member of the opioid receptor family, but, despite sharing a common signal transduction pathway with the ‘classical’ opioid receptors (mu:μ; delta:δ; kappa:κ), endogenous or exogenous agonists or antagonists for classical receptors do not display meaningful affinity [5]. The endogenous ligand for NOP, N/OFQ, similarly has little to no affinity for the classical opioid receptors. Upon activation, NOP receptors couple to G_i/o_ proteins leading to a decrease in cyclic Adenosine Monophosphate (cAMP) production, activation of inwardly-rectifying potassium channels, closure of calcium channels and activation of mitogen-activated protein kinase pathways (MAPK) including ERK 1/2 [5–7]. NOP receptors have been found throughout the central nervous system, as well as in cardiovascular and immune systems. It is this activity in the latter systems that draws focus to a role in sepsis and septic shock.

NOP and N/OFQ mRNA transcripts have been identified in a large subset of polymorphonuclear cells, with surface expression of NOP identified on these cells through use of a NOP-specific fluorescent ligand, N/OFQ_ATTO594_ [7–9]. Furthermore, N/OFQ has been shown to directly influence inflammatory responses, with N/OFQ acting in a modulatory capacity for the chemo-attraction of leucocytes by mast cells [9].

A link between increased N/OFQ in sepsis and the development of hypotension is supported by several studies in animals. For example in rats, Rizzi and colleagues showed that intravenous N/OFQ and UFP-112 (a longer lasting NOP agonist) reduced mean arterial pressure and caused a bradycardia [10]. Moreover, Burmeister et al., also demonstrated hypotension, bradycardia and diuresis in mice after intracerebroventricular administration of N/OFQ [11]. The inflammatory mediator LPS induces dilation of the rat microvasculature, seen during sepsis, which is enhanced by the presence of N/OFQ and reduced by UFP-101, further indicating a role for NOP in the onset of hypotension in sepsis [12]. Moreover, in a caecal ligation-puncture model of sepsis in the rat, N/OFQ reduced and UFP-101 improved survival [13].

Mediators of immune function may cause vasodilatation and hypotension in sepsis by effects on the vascular endothelium. Human umbilical vein endothelial cells (HUVEC) have been identified as an *in vitro* model to study immune-vascular interactions in sepsis [14,15]. In this study we have used freshly isolated HUVEC and human vascular smooth muscle cells (HVSMC) prepared from human umbilical cords to examine NOP expression and the effects of lipopolysaccharide (LPS) and peptidoglycan (PepG) as an *in vitro* sepsis mimic. We used cells with limited passage to avoid de-differentiation [16]. In the presence and absence of LPS and PepG we measured NOP mRNA by quantitative PCR, NOP receptor expression using the NOP fluorescent probe, N/OFQ_ATTO594_ and NOP function by measuring N/OFQ-induced MAPK activation.

## Methods

### Tissue collection and culture

Umbilical cord tissue samples were provided by the Anthony Nolan Trust, who obtained ethical approval (East Midlands-Derby, UK Research Ethics Committee) and written informed consent from the donor’s parents. HUVECs (n = 50) were isolated from the umbilical vein using collagenase digestion as described previously [17]. Briefly, cannulated veins were sealed with a clamp and washed with PBS, followed by flushing with air before addition of collagenase solution (0.5mg/ml). The tissue sample was massaged and incubated at 37°C for 15 minutes. The collagenase solution, containing the HUVEC cells, was collected and the vein washed with PBS to collect any remaining cells. The cell suspension was centrifuged for 5 minutes at 500x*g* and the cell pellet was transferred to a 0.2% gelatin-coated T75 flask and incubated at 37°C/5% CO_2_ in HUVEC growth media M199 supplemented with 10% foetal calf serum (FCS), 1% penicillin/streptomycin, 10U/ml heparin, 5ng/ml human FGF-acidic, 2.5μg/ml thymidine and 4.5μg/ml endothelial cell growth supplement.

Using the same umbilical cord tissue samples as mentioned previously, HVSMC cells were isolated from the umbilical arteries using a previously reported method [18]. Briefly, the umbilical arteries were dissected out of the umbilical cord and the adventitia removed. The arteries were then cut into small pieces (<5mm) and placed into 0.2% gelatin-coated T25 flasks. The tissue explants were incubated at 37°C for 2 hours to ensure adhesion of the tissue to the flask. After this, media was added comprising DMEM with GlutaMAX (Gibco, UK) supplemented with 15% FCS, 1% penicillin/streptomycin, 0.5ng/ml human EGF, 5μg/ml human insulin and 2ng/ml human FGF-basic. After the HVSMCs had migrated out of the tissue explants, these were removed and the cells were cultured. At passage 2 onwards, the cells were cultured in media as above, except with 10% FCS concentration.

In some experiments HUVEC and HVSMC (≤ passage 4) were incubated in the absence and presence of 100ng/ml LPS and 100ng/ml peptidoglycan (PepG), with concentrations chosen based on previous studies [19].

### RNA extraction and PCR

Both HUVEC and VSMC were grown in 6 well plates and, once reaching 80% confluency, selected wells were used for experimentation. HUVEC were incubated with 100ng/ml LPS and 100ng/ml PepG for either 24 or 48 hours (at 37°C) alongside untreated controls. Following this incubation period, cells were washed with phosphate buffer solution and 1ml Tri-reagent® was added. Samples were stored in 1ml aliquots at -80°C. Total RNA was extracted (8), with the final pellet re-suspended in PCR grade water. A NanoDrop 2000 (LabTech) was used to determine RNA mass, with purity assessed from 260/280nm ratio of >1.8. Isolated RNA was processed using a TURBO DNA-free® kit to remove genomic DNA contamination. RNA was reverse transcribed to produce cDNA using a High Capacity Reverse transcription kit (Thermo Fisher Scientific, UK). NormFinder algorithm indicated the most stable housekeeper combination was B2M (for HUVEC and HVSMC) and either ELF2B1 (HUVEC) or ELF1 (HVSMC); these were included in all QPCR experiments [8,20].

Quantitative PCR was used to assess mRNA quantity. Commercially available NOP receptor (Hs00173471_m1) Taqman® gene expression assays from Applied Biosystems were used to measure levels of NOP mRNA and the chosen housekeeper mRNA, B2M and either ELF2B1 (HUVEC) or ELF1 (VSMC). A StepOne instrument (Applied Biosystems) was used with a qPCR thermal profile of 2min at 50°C, 10 min at 95°C, 50 cycles of 15s at 95°C and 1 min at 60°C. Non-template controls were included for all samples.

Data is expressed as cycle threshold (Ct—with each cycle representing a doubling of initial mass) relative to the geometric mean of B2M and either ELF2B1 or ELF1; ΔC_t_.

### Confocal microscopy

In all confocal experiments, coverslips (28mm Menzel glaser #1-coverslips, Thermo-Fisher) were ethanol-sterilised, and treated with CellTak™ (1μg.ml^-1^) (Sigma, UK). Use of coverslips is a requirement for the perfusion system where live cells are required to be maintained at low temperatures for fluorophore binding (see below and [7]). Upon reaching confluence, HUVECs or HVSMCs were passaged onto treated coverslips and either incubated in media containing 100ng/ml LPS and 100ng/ml PepG for 24 and 48hr (HUVEC only), or in media alone. Following this incubation, cells were washed with Krebs buffer, pH 7.4, at 4°C, with temperature maintained using a PDMI-2 temperature controller and TC202A micro-incubator (Burleigh, Digitimer, Cambridge, UK) [7].

N/OFQ_ATTO594_, a highly selective (red) fluorescent ligand for the NOP receptor [7], was added onto coverslips at a concentration of 100nM. This concentration was based on characterisation in recombinant systems where binding mirrored [^3^H]N/OFQ binding [7]. Images were captured using a Nikon Eclipse C1Si microscope (Surrey, UK), using an oil immersion 60x objective. N/OFQ_ATTO594_ was incubated with the cells for 5 minutes at 4°C, following which unbound was cleared by perfusion with 4°C Krebs buffer. As we previously reported this low temperature eliminates receptor internalisation [7]. Cells were sequentially imaged at 594nm for N/OFQ_ATTO594_ and 488nm, for Hoechts live cell nuclear dye (blue). Specific binding of N/OFQ_ATTO594_ was determined through use of the NOP antagonist, SB-612111 (1μM) [21]. SB-612111 was pre-incubated with cells for 15 minutes before addition of 100nM N/OFQ_ATTO594_ [7]. In all experiments, samples were tested in duplicate. Images were processed and analysed using ImageJ. As a positive control for binding we have included Human Embryonic Kidney cells stably expressing the human NOP receptor; these cells were cultured as described in [22] and used as above.

### Western blotting

Functional NOP receptor expression was determined by measuring ERK1/2 phosphorylation in both HUVEC and HVSMC using Western blotting; this assay was selected as it requires relatively small amounts of tissue and produces an easily quantified readout. HUVEC or HVSMC were grown in 6-well plates until 70% confluent. Cells were then serum starved for 24hrs and variably treated with 100ng/ml LPS and 100ng/ml PepG for 24 or 48hours. Next cells were incubated in 37°C Krebs buffer for 30 minutes then challenged with 1μM N/OFQ for up to 30 minutes. Upon completion of the time course, the assay was terminated by removal of Krebs buffer, rinsing with ice-cold PBS and addition of ice-cold lysis buffer [22]. Cells were scraped, lysis buffer was collected in 1ml tubes, incubated on ice for 10 minutes, and spun at 17000xg at 4°C for 15 minutes. The supernatant was removed and added to an equal volume of 2xLamelli buffer, after which, samples were denatured at 95°C for 5 minutes [22].

Following denaturation, proteins were separated on 10% SDS-PAGE, transferred onto nitrocellulose paper by wet transfer and blocked in 10% milk TBS-T solution (150mM NaCl, 20mM Tris HCl, 0.05% Tween 20; pH 7.5) for 2 hr at room temperature under gentle agitation. Membranes were subsequently incubated in primary phospho-ERK1/2 antibodies (1:6000 dilution; #4377S, Cell Signalling Technology) and loading control antibody; vinculin (1:1200; #4650S, Cell Signalling Technology), diluted in TBS-T overnight at 4°C. Primary antibody was removed and membranes washed 6 times, for 5 minutes per wash, in TBS-T. Membranes were incubated in HRP-conjugated secondary antibodies (1hr room temperature; 1:1000 dilution 5% milk/TBS-T solution; #7075, Cell Signalling Technology). Chemiluminescence detection, using the ChemiDoc™ MP imaging system (Bio-Rad, UK) was used to visualise immune-reactive bands. Blots were analysed using the ImageLab software (bio-rad, UK). All data is corrected for loading (Density of ERK/Density of Vinculin) and then measured as a ratio over basal activity [22].

### Data analysis

All data are presented as mean±SEM from (n) experiments as noted in the results and Figure/Table legends. Confocal images are representative of (n) as noted. In HUVEC experiments PCR and confocal microscopy was performed in cell lines from 50 patients and 20 were analysed in Western blotting. In VSMC experiments PCR and confocal microscopy was performed in cell lines from 10 patients and 8 were analysed in Western blotting. The reduced number was selected as there was no LPS/PepG inducible signal (**see** **results**). Statistical analysis is described in the relevant Table and Figure legends and *P*-values ≤0.05 were considered statistically significant.

## Results

### HUVEC

#### PCR

Messenger RNA extracted from primary cultured HUVECs was used in qPCR to probe for NOP expression. All samples produced robust amplification of the housekeeper genes B2M and ELF2B1. In control conditions, NOP mRNA was present in all 50 cell lines tested demonstrating a mean ΔC_t_ of 6.25. Of the cell lines treated with 100ng/ml of LPS/PepG, 49 also expressed NOP mRNA. In a single subject HUVEC cell line, termed PN5, LPS/PepG exposure led to cell death. In the remaining 49 cell lines 24 hours exposure to LPS/PepG decreased NOP mRNA with a mean ΔC_t_ of 8.73. This trend continued after 48 hours, with a ΔC_t_ of 8.98. Both ΔC_t_ values obtained at 24hours and 48 hours were significantly different from ΔC_t_ obtained from control HUVEC samples (ANOVA and Bonferroni p<0.05) (**Table 1**). These changes at 24 and 48 hours equated to an approximate 80% decrease in NOP mRNA expression.

**Table 1:** NOP mRNA from HUVEC and HVSMC was measured in qPCR experiments relative to two housekeeper genes, B2M and either EFL2B1 (HUVEC) or ELF1 (HVSMC), to determine ΔC_t_ relative to the housekeeper geometric mean.

	Control			24hr LPS/PepG Treatment			48hr LPS/PepG Treatment		
	HK Ct (Geom)	NOP Ct	ΔCt Geom	HK Ct (Geom)	NOP Ct	ΔCt Geom	HK Ct (Geom)	NOP Ct	ΔCt Geom
**HUVEC**	23.69(±0.24)	29.94(±0.28)	6.25(±0.17)	23.71(±0.24)	32.45(±0.31)	8.73(±0.18)*	22.19(±0.28)	31.17(±0.36)	8.98(±0.17)*
**HVSMC**	23.08(±2.72)	29.58(±1.76)	6.94(±2.04)	23.16(±0.90)	28.99(±1.21)	6.75(±0.67)			

In HUVEC, treatment with 100ng/ml LPS/PepG led to significant increases in ΔC_t_ values for NOP; this is a decline in expression. For VSMC, 24hr treatment with 100ng/ml LPS/PepG did not induce any significant change in mRNA production. For HUVEC, data are the mean±SEM of 50 cell lines for control and 49 for LPS/PepG treated. For HVSMC, data are the mean±SEM of 10 cell lines for control and 10 experiments for 24hr LPS/PepG treated. For HUVEC; *p<0.05 (ANOVA and Bonferroni post hoc analysis) represents changes when compared with the control. For HVSMC, a Student’s t-test showed no significant difference between control and LPS/PepG treated cell lines.

#### Confocal microscopy

**Fig 1:**
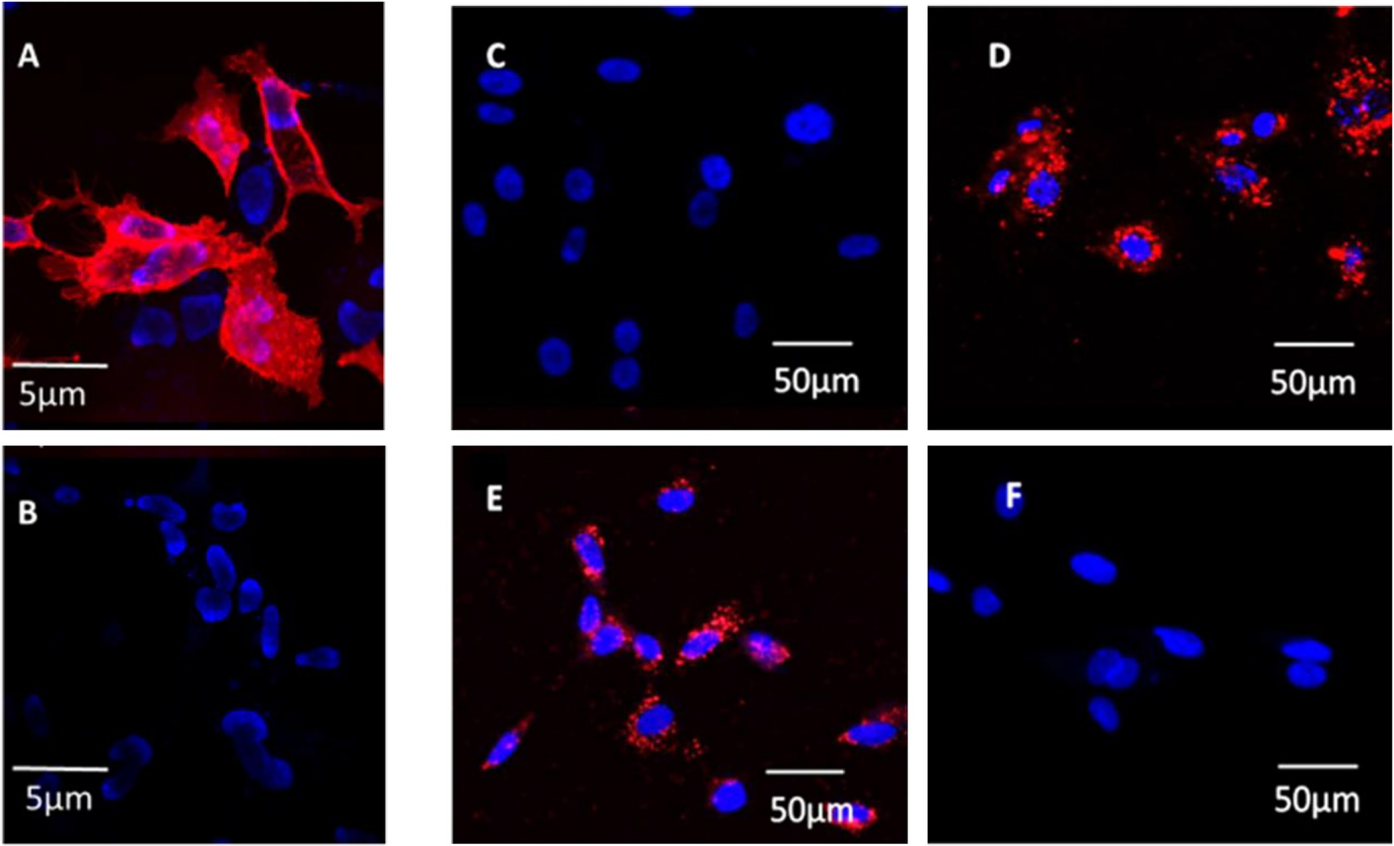
N/OFQ_ATTO594_ binding. HEK_hNOP_ cells (positive control) loaded with Hoechts live cell nuclear dye (Blue) were incubated with 100nM N/OFQ_ATTO594_ this bound (A) in a SB-612111 sensitive manner (B). HUVEC also loaded with Hoechts dye (Blue) failed to bind 100nM N/OFQ_ATTO594_ (C). N/OFQ_ATTO594_ bound to the surface of HUVEC treated with 100ng/ml LPS and 100ng/ml PepG for 24 hours (D) and 48 hours (E). Binding of N/OFQ_ATTO594_ was prevented by 1μM SB-612111 (F; illustrated at 24 hour time point). Images are representative of 49 individual cell lines with experiments carried out in duplicate.

**Fig 2:**
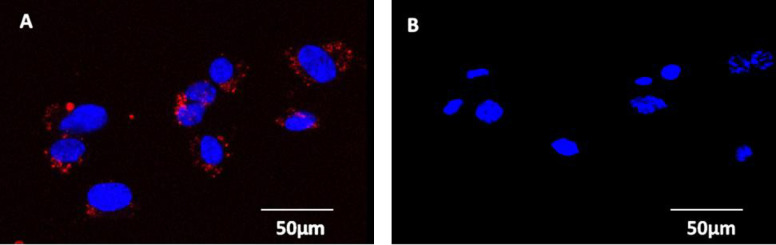
(A) Untreated (LPS/PepG) HUVEC PN5 cell line binds 100nM N/OFQ_ATTO594_ and this binding is blocked in the presence of 1μM SB-612111 (B). Images are representative of experiments repeated 5 times.

Results from PCR demonstrated the presence of NOP mRNA in all 50 HUVEC cell lines, however the presence of mRNA does not necessarily equate to surface expression of a functional receptor. In order to determine the presence of NOP receptors the NOP-specific fluorescent ligand, N/OFQ_ATTO594_, was used. As a positive control N/OFQ_ATTO594_ bound to the surface of HEK_hNOP_ cells (**Fig 1A**) and this was blocked by the NOP antagonist SB-612111 (**Fig 1B**) In 49 individual control HUVEC cell lines, 100nM N/OFQ_ATTO594_ failed to bind to untreated (100ng/ml of LPS/PepG) cells (**Fig 1C**). In these 49 cell lines, treatment at both 24hrs (**Fig 1D**) and 48hrs (**Fig 1E**) with 100ng/ml LPS and 100ng/ml PepG treatment, led to binding of N/OFQ_ATTO594_. Binding of N/OFQ_ATTO594_ in these samples is blocked by the NOP-selective antagonist SB-612111 (1μM) **(Fig 1F**). In PN5 cells, 100nM N/OFQ_ATTO594_ was able to bind to control samples (**Fig 2A**) and could be blocked by SB-612111 (n = 5) (**Fig 2B**). As noted above 100ng/ml of LPS/PepG treatment caused cell death in this line.

**Fig 3:**
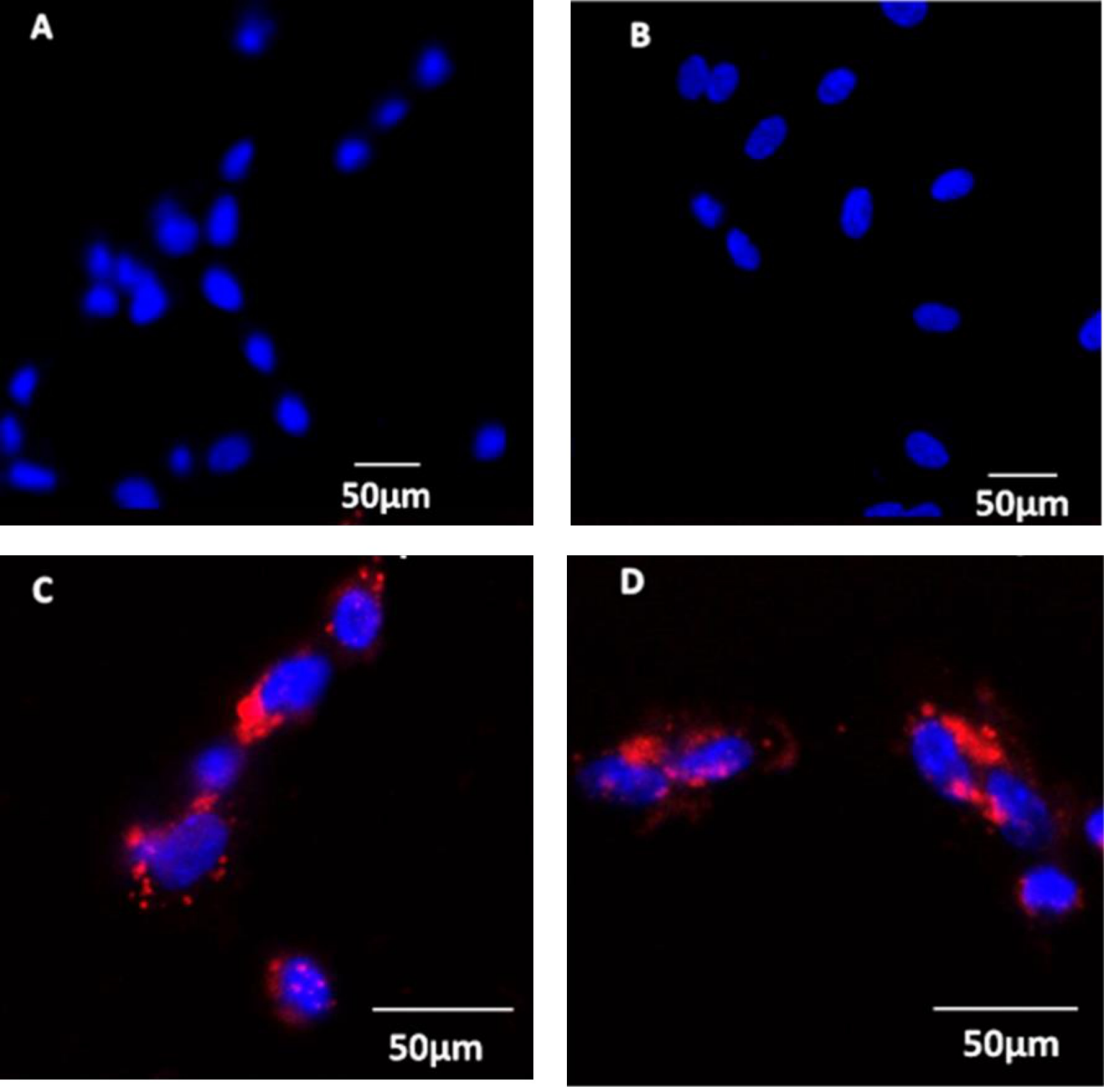
To determine the effect of medium-term culturing of HUVEC on NOP expression, HUVEC cell line PN8 (which required 100ng/ml LPS/PepG treatment to demonstrate NOP expression) was cultured alongside HUVEC cell line PN5 (which demonstrates expression independent of LPS/PepG treatment) and probed with 100nM N/OFQ_ATTO594_. In this example at passage 3 (without LPS/PepG treatment), N/OFQ_ATTO594_ failed to bind to PN8 (A) but demonstrated binding to PN5 (C). After 20 passages, untreated PN8 (B) again failed to bind N/OFQ_ATTO594_, while PN5 (D) again demonstrated binding.

Many studies using primary cultured HUVECs are with older passages. To determine whether long term culture changes NOP expression, PN5 (which expresses NOP in the absence of LPS/PepG) and PN8 (a cell line that required LPS/PepG treatment to induce NOP expression) were maintained over 20 passages with expression probed (**Fig 3**). In both instances, PN8 (**Fig 3A & 3B**) and PN5 (**Fig 3C & 3D**) demonstrated no change in their respective expression, with PN5 maintaining NOP expression from passage 2 (**Fig 3C**) through to passage 22 (**Fig 3D**).

There was no correlation between change in mRNA and N/OFQ_ATTO594_ binding (**S1 Fig**).

#### Western blot

**Fig 4:**
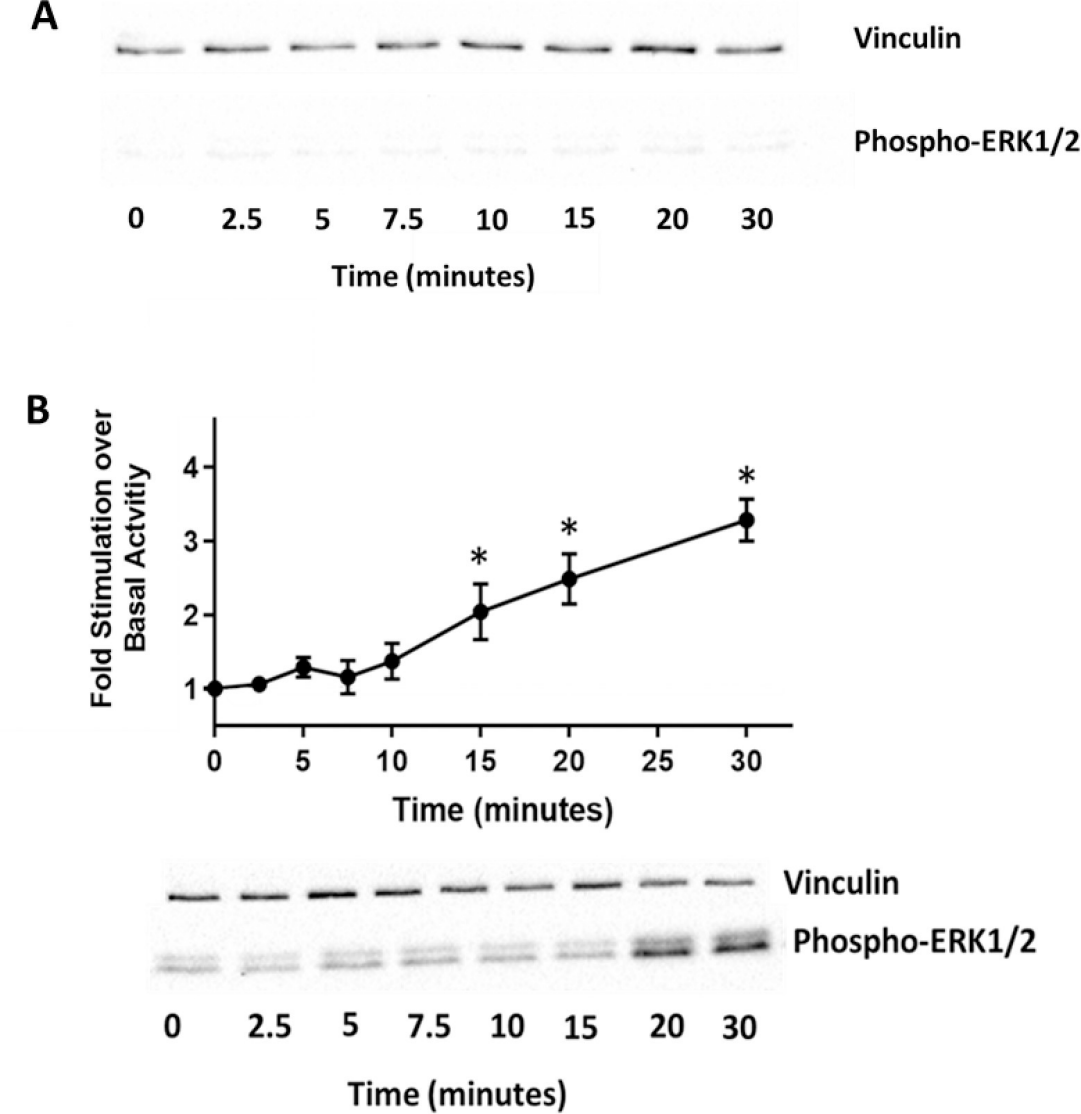
ERK1/2 Phosphorylation profiles. A) Unstimulated HUVEC displayed no activity after incubation with 1μM N/OFQ. B) Stimulation of ERK1/2 in LPS/PepG treated HUVEC by 1μM N/OFQ was measured over 30 minutes. Representative Western blots are shown below the graph. Data are the mean±SEM from 20 separately cultured lines. Analysis was undertaken on raw data using ANOVA and Bonferroni post hoc analysis, *statistically greater than raw basal activity (p<0.05).

**Fig 5:**
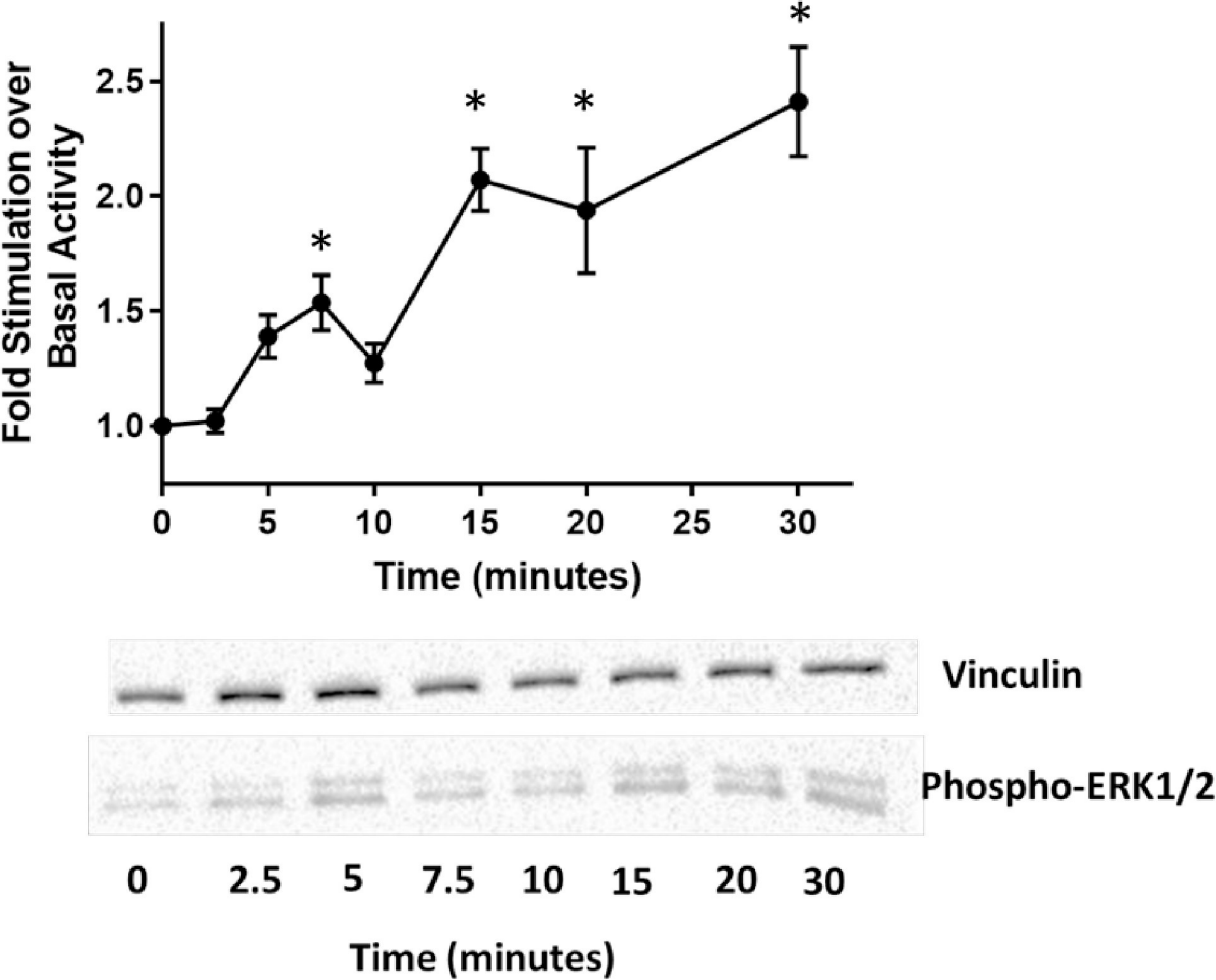
ERK1/2 Phosphorylation profile. In the HUVEC line PN5 that expresses NOP receptors in the **absence** of LPS/PepG treatment, addition of 1μM N/OFQ leads to stimulation of ERK1/2. Representative Western blots are shown below the graph. Data are the mean±SEM of 5 separate experiments. Analysis was undertaken on raw data using ANOVA and Bonferroni post hoc analysis, *statistically greater than raw basal activity (p<0.05).

Activation of HUVEC NOP receptors was measured by determining activation of the ERK1/2 pathway. When measuring activity in untreated control samples, 20 cell lines showed no activation over a period of 30 minutes when incubated with 1μM N/OFQ (**Fig 4A**). This is consistent with the lack of surface receptor protein demonstrated with N/OFQ_ATTO594_. When incubated for 24hr with 100ng/ml LPS/ PepG, N/OFQ-induced activation of ERK1/2 was seen after 10 minutes and continued to increase up to 30 minutes (**Fig 4B**). In the HUVEC cell line, PN5, activation of ERK1/2 was seen in the untreated control sample, with activation beginning at 5 minutes and continuing through to 30 minutes post N/OFQ incubation (n = 5) (**Fig 5**). Measurement of ERK1/2 activity in PN5 HUVEC treated with 100ng/ml LPS/ PepG was not possible due to cell death. **Full blots are provided in the S1 Raw data.**

### HVSMC

#### PCR

Messenger RNA extracted from primary cultured HVSMC was used in qPCR to probe for NOP expression. All cell lines produced robust amplification of the housekeeper genes B2M and ELF1. In control untreated samples, NOP mRNA was present in all 10 cell lines tested with a mean ΔC_t_ of 6.94 (**Table 1**). All 10 cell lines were treated with 100ng/ml of LPS/PepG for 24 hour and there was no change in NOP mRNA; ΔC_t_ 6.75. Due to the lack of an inducible signal we limited the number of cell lines to 10.

#### Confocal microscopy

**Fig 6:**
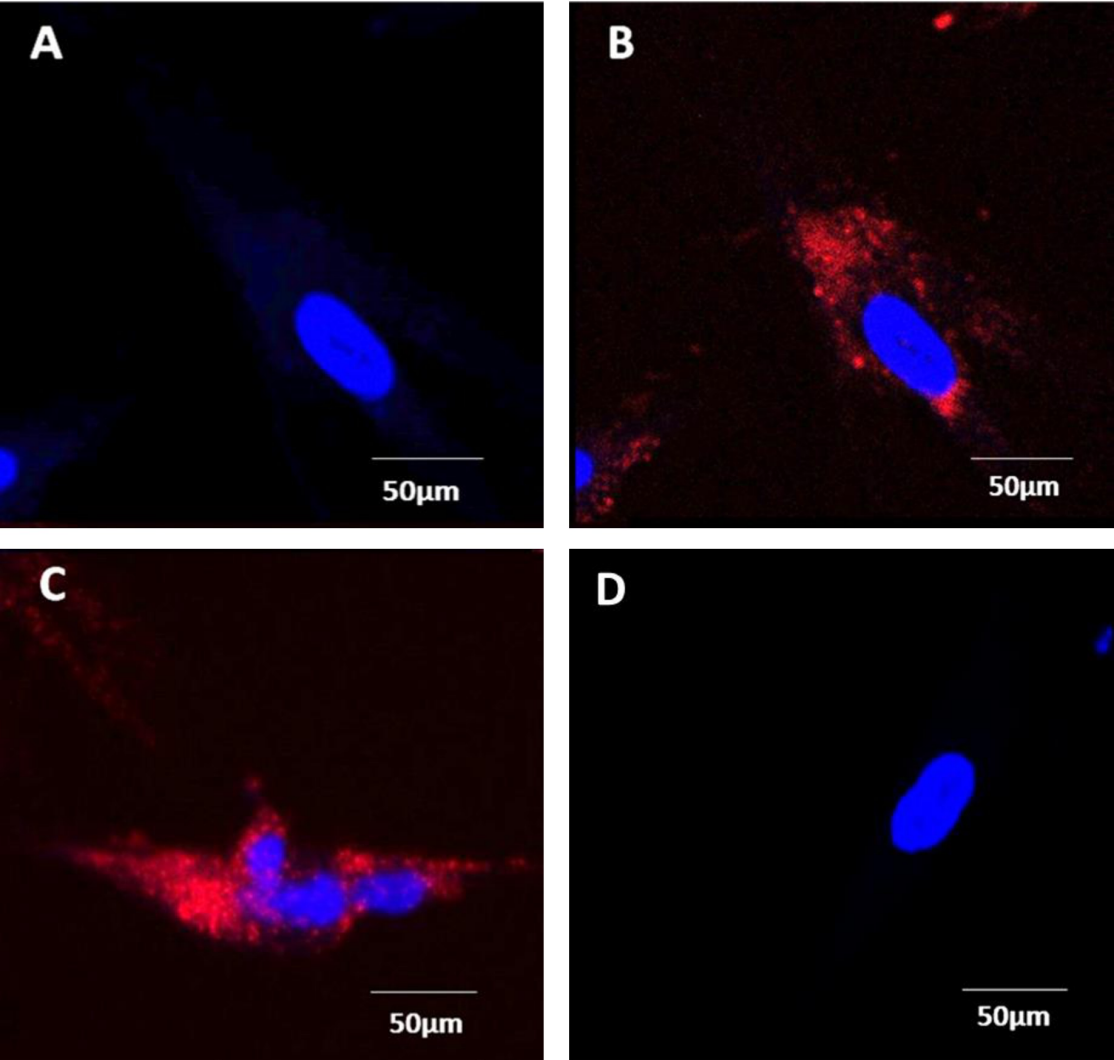
HVSMC (A) loaded with Hoechts live cell nuclear dye (Blue) were incubated with 100nM N/OFQ_ATTO594_ (B) which bound to the cell surface, with binding of N/OFQ_ATTO594_ remaining unchanged in HVSMCls treated with 100ng/ml LPS/ PepG for 24 hours. Binding of N/OFQ_ATTO594_ was prevented by 1μM SB-612111 (D). Images are representative of 10 individual cell lines with experiments carried out in duplicate.

All ten cell lines (Hoechts stained **Fig 6A**) were subsequently tested for N/OFQ_ATTO594_ binding in confocal microscopy. In untreated samples N/OFQ_ATTO594_ bound to the cell surface of VSMC (**Fig 6B**), with binding unaffected by 24hr incubation with 100ng/ml LPS/PepG (**Fig 6C**). Binding of N/OFQ_ATTO594_ was blocked by the NOP-selective antagonist SB-612111 (1μM) (**Fig 6D**).

#### Western blot

**Fig 7:**
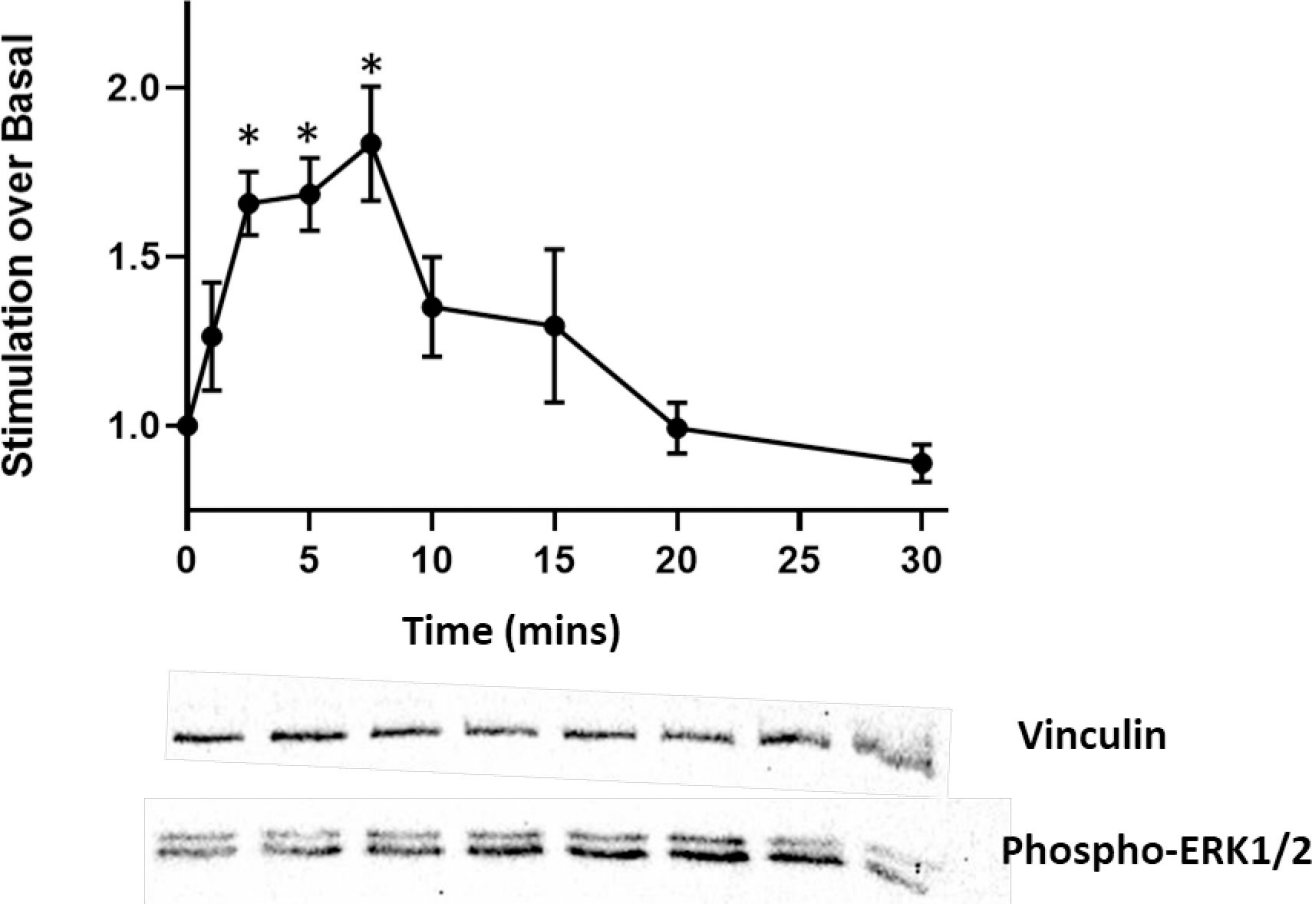
In HVSMC, NOP receptor induced activation of ERK1/2 following stimulation with 1μM N/OFQ. Representative Western blots shown below the graph. Analysis was undertaken on raw data using ANOVA and Bonferroni post hoc analysis, *statistically greater than raw basal activity (p<0.05).

Activation of HVSMC NOP receptors was measured by determining activation of the ERK1/2 pathway. N/OFQ (1μM) increased ERK1/2 phosphorylation after 2.5 minutes, peaking between 7.5–10 minutes, before returning to basal levels at 20 minutes (**Fig 7**). This series of experiments were performed on 8 cell lines. **Full blots are provided in the S1 Raw data.**

## Discussion

In freshly isolated HUVEC, the presence of NOP mRNA was detected in all cell lines tested. When challenged with an *in vitro* sepsis mimic of a combination of LPS and PepG for 24–48 hrs, NOP mRNA declined. It is tempting to suggest that the reduction of mRNA was a consequence of its being used to produce functional receptor protein as shown with our novel NOP ligand; N/OFQ_ATTO594_ [7]. Endothelial cell NOP mRNA was therefore only translated into protein by stimulation with LPS and PepG and these receptors were functionally active as stimulation with N/OFQ enhances ERK1/2 phosphorylation. In HVSMC NOP mRNA was expressed with or without an *in vitro* sepsis challenge. Moreover, NOP was expressed at the cell surface and this was also functionally active in that there was a robust ERK1/2 phosphorylation signal. We have used ERK1/2 phosphorylation as a sensitive and relatively easy to measure marker of receptor function, particularly where expression is predicted to be low. In our study the ERK1/2 signal peaked earlier in muscle compared to endothelium.

While the presence of NOP mRNA was detected in all cell lines, the presence of surface NOP receptor was found in only one (PN5) of the 50 untreated HUVEC cell lines. However, following incubation with LPS/PepG for both 24 and 48 hours NOP receptor was detected on the surface of these treated HUVEC via binding of the fluorescent probe N/OFQ_ATTO594_. That this binding was on the cell surface is confirmed by the observation that N/OFQ_ATTO594_ binding was inhibited by the NOP-selective antagonist SB-612111 (indicating selective binding of the probe to NOP receptor) with neither the probe (large peptide) nor the antagonist being able to cross the plasma membrane. Moreover, as the experiments were performed at 4°C agonist driven NOP internalisation will not occur; we have confirmed this in high expressing recombinant systems [7]. Interestingly, the PN5 cell line, which expressed NOP in control conditions, did not survive in all LPS/PepG incubation experiments. This is not seen in cells where induction increased expression, especially in 48 hour treatment where after only 24 hours we know there is significant NOP expression. Long-term culture (>20 passages) of HUVECs did not lead to any changes in expression of NOP receptor indicating that from a NOP-functional perspective there was no short term dedifferentiation.

In 49 HUVEC cell lines, phosphorylation of ERK1/2 was used to detect functional activity following LPS/PepG induced NOP surface expression. In these samples, 1μM N/OFQ produced phosphorylation of ERK1/2 after 10 minutes, with ERK1/2 activity remaining elevated for the remainder of the 30 minute assay. In the untreated PN5 sample, N/OFQ produced an initial response after 5 minutes. This increased, throughout the course of the 30 minute assay.

In freshly isolated VSMC, NOP mRNA was detected in all cell lines tested, this was unaffected by LPS/PepG treatment (for 24 hours) and there was no adverse consequences in terms of survival. When untreated VSMC were probed with N/OFQ_ATTO594_, binding of the ligand to the cell surface was detected; this was totally blocked by the NOP antagonist SB-612111. These cell surface receptors were also coupled to ERK1/2 but the response was different to that seen in HUVECs. The very early response in muscle might suggest a G-protein driven event while the later response in the endothelium might suggest an arrestin driven event [23]; this will require rigorous experimental evaluation. Delayed activation of ERK1/2 may also be associated with localisation to the nucleus, resulting in interaction with growth factor-induced genes possibly affecting cell proliferation and differentiation [24].

In this study a single cell line was shown to express NOP without the need for LPS/PepG stimulation. This cell line (termed PN5) displayed several distinct features not seen in the remainder of the HUVECs tested. Activation of ERK1/2 occurred earlier but was still different to that seen in muscle. The most striking difference was the sensitivity (cell death) to LPS/PepG challenge. Our ethics approval does not allow detailed collection of patients’ clinical or personal information so it is not possible to look back at pre-existing pathology, clinical course or concurrent medications for this subject. In a series of immunohistochemical analyses (**S2 Fig**) we have confirmed that PN5 is HUVEC in origin; it was possible that this line was muscle that we know expresses NOP independent of LPS/PepG treatment. Our incidence of 1 in 50 compared favourably with incidence of post-partum infection at 3.9% [25]; sepsis would be lower. It is tempting to speculate that this delivery was associated with infection but as noted our ethics approval does not allow this to be addressed.

HUVEC have long been used in cardiovascular [26] and more specifically in sepsis [27] research. Early passage primary HUVEC are superior to older and more established cell lines in that their physiology likely remains similar to that of the endothelium post collection [28–30]. The major disadvantages of cultured HUVEC include loss of primary characteristics from between 6–10 passages onwards [31]. Similarly freshly isolated early passage VSMC are highly differentiated and closely represent the *in vivo* situation [32]. Indeed, surface protein expression on VSMCs is similar to *in vivo* smooth muscle [33]. As with HUVEC, long-term passaging raises the possibility of dedifferentiation [34].

As stated previously, the combination of LPS and PepG provide a useful *in vitro* model for sepsis and this combination of endotoxins have been shown to cause significant inflammatory responses in HUVEC [14,15] as well as VSMC [35,36]. The expression profile in our primary HUVEC cells is markedly different from a previous paper, where NOP was present and functional on the HUVEC surface [37]. The authors indicate that they have used “Commercial human umbilical vein endothelial cells”, age, passage number and number of lines used was not stated so it is difficult to reconcile these data with ours.

Pulling our data set together, we have demonstrated NOP upregulates in the endothelium, immune cells are recruited to the site of infection and we have recently demonstrated that immune cells release N/OFQ [38]. Given that (in experimental animals) NOP activation with exogenous N/OFQ causes blood vessel dilation and a fall in blood pressure it is reasonable to suggest that immune derived N/OFQ is a hypotensive agent in this context. We have shown that N/OFQ activates MAPK in LPS/PepG treated endothelial cells and this is capable of producing nitric oxide that will then be the final mediator of vasorelaxation [39]. Two pieces of information require further comment. First, work from Champion in 1998 in the rat showed L-NAME sensitivity for MOP opioid receptor activation with Endomorphin but not NOP activation with N/OFQ [40]. There are clearly species differences to consider. Second, in the absence of LPS/PepG VSMC already express functional NOP and activation increases ERK1/2 phosphorylation with the potential to modulate the contractile response. We believe the primary source of N/OFQ is immune in origin and distributed within the vasculature so in this context the endothelium is the primary target.

What does this all mean for N/OFQ-NOP, sepsis and sepsis induced hypotension? There is good evidence for a NOP signal in sepsis. In caecal-ligation and puncture model in rats N/OFQ worsens, and the NOP antagonist UFP-101 improves survival [13]. Moreover, N/OFQ concentrations are increased in human patients with sepsis [20]. It will be interesting to study NOP antagonism as an adjunct to existing therapy in the intensive care unit. In recent developments such a molecule is in clinical trials; BTRX-246040. This NOP antagonist is being trialled in major depressive illness (NCT03193398) and Parkinson’s disease (NCT03608371) where NOP antagonists have efficacy. Importantly the depressive illness trial has some data where there was one serious adverse event in the placebo group and minor adverse event rates in both BTRX-246040 and placebo groups. It will be interesting to trial this molecule in patients with sepsis.

## Supporting information

S1 Fig No correlation between NOP mRNA (PCR) and NOP receptor protein (N/OFQ_ATTO594_ binding). HUVEC NOP mRNA and NOP receptor protein was measured by qPCR and N/OFQ_ATTO594_ binding respectively as in the main methods. In HUVEC lines treated with 100ng/ml LPS/PepG (needed to translate NOP mRNA into protein) there was no correlation between message and receptor. Based on N/OFQ_ATTO594_ binding (inset) expression peaked after 24 hours (there was a small but statistically significant difference between 24 and 48 hour groups (*p = 0.03, paired t-test, n = 49, 5.6% increase at 48 hours).
(DOCX)

S2 Fig HUVEC-PN5 line is endothelium and not muscle. Immunofluorescence Analysis of HUVEC cell Line PN5. In order to confirm the cell type identity of line PN5, immunofluorescence for the endothelial marker von-Willebrand Factor (vWF), the smooth muscle cell marker α-smooth muscle actin (α-SMA) and the fibroblast marker TE-7 with smooth muscle cell (human umbilical cord artery cells; HVSMC) and fibroblast (MRC5) positive controls. A secondary antibody-only control was included for all cell types. Green fluorescence indicates positive staining for the indicated marker. Nuclei were counterstained with Hoechst 33342 (blue). Images were obtained using with an Operetta CLS high-content imaging system (Perkin Elmer) using a 10x objective lens. Scale bar = 200μm.
(DOCX)

S1 Raw data (PDF)
