# Supplementary material for: In vitro sepsis induces Nociceptin/Orphanin FQ receptor (NOP) expression in primary human vascular endothelial but not smooth muscle cells
Source: PLoS One. 2022 Sep 15;17(9):e0274080. doi: 10.1371/journal.pone.0274080 (PMC9477356; doi:10.1371/journal.pone.0274080)
Supplement: S1 Fig — HUVEC NOP mRNA and NOP receptor protein was measured by qPCR and N/OFQATTO594 binding respectively as in the main methods. In HUVEC lines treated with 100ng/ml LPS/PepG (needed to translate NOP mRNA into protein) there was no correlation between message and receptor. Based on N/OFQATTO594 binding (inset) expression peaked after 24 hours (there was a small but statistically significant difference between 24 and 48 hour groups (*p = 0.03, paired t-test, n = 49, 5.6% increase at 48 hours). (DOCX) [file pone.0274080.s001.docx]

***In vitro* sepsis induces Nociceptin/Orphanin FQ receptor (NOP) expression in primary human vascular endothelial but not smooth muscle cells.**

Mark F. Bird^1^, Barbara Gallacher-Horley^1^, John McDonald^1^, David G. McVey^1^, Fatin Al-Janabi^1^, Remo Guerrini^2^, Girolamo Calo^3^, Shu Ye^1^, Jonathan P. Thompson^1^ and David G. Lambert^1^

^1^Department of Cardiovascular Sciences, Anaesthesia, Critical Care and Pain Management, University of Leicester, Hodgkin Building, Leicester, LE1 9HN. UK.

^2^Department of Chemical, Pharmaceutical and Agricultural Sciences, University of Ferrara, 44121 Ferrara, Italy.

^3^Department of Pharmaceutical and Pharmacological Sciences, University of Padova, 35131 Padova, Italy.

**SUPPORTING INFORMATION; SUPPLEMENT.**

**FIGURE-1**

**No correlation between NOP mRNA (PCR) and NOP receptor protein (N/OFQ_ATTO594_ binding).**

**Supplement Figure-1:** HUVEC NOP mRNA and NOP receptor protein was measured by qPCR and N/OFQ_ATTO594_ binding respectively as in the main methods. In HUVEC lines treated with 100ng/ml LPS/PepG (needed to translate NOP mRNA into protein) there was no correlation between message and receptor. Based on N/OFQ_ATTO594_ binding (inset) expression peaked after 24 hours (there was a small but statistically significant difference between 24 and 48 hour groups (*p=0.03, paired t-test, n=49, 5.6% increase at 48 hours).
