# Supplementary material for: In vitro sepsis induces Nociceptin/Orphanin FQ receptor (NOP) expression in primary human vascular endothelial but not smooth muscle cells
Source: PLoS One. 2022 Sep 15;17(9):e0274080. doi: 10.1371/journal.pone.0274080 (PMC9477356; doi:10.1371/journal.pone.0274080)
Supplement: S2 Fig — Immunofluorescence Analysis of HUVEC cell Line PN5. In order to confirm the cell type identity of line PN5, immunofluorescence for the endothelial marker von-Willebrand Factor (vWF), the smooth muscle cell marker α-smooth muscle actin (α-SMA) and the fibroblast marker TE-7 with smooth muscle cell (human umbilical cord artery cells; HVSMC) and fibroblast (MRC5) positive controls. A secondary antibody-only control was included for all cell types. Green fluorescence indicates positive staining for the indicated marker. Nuclei were counterstained with Hoechst 33342 (blue). Images were obtained using with an Operetta CLS high-content imaging system (Perkin Elmer) using a 10x objective lens. Scale bar = 200μm. (DOCX) [file pone.0274080.s002.docx]

***In vitro* sepsis induces Nociceptin/Orphanin FQ receptor (NOP) expression in primary human vascular endothelial but not smooth muscle cells.**

Mark F. Bird^1^, Barbara Gallacher-Horley^1^, John McDonald^1^, David G. McVey^1^, Fatin Al-Janabi^1^, Remo Guerrini^2^, Girolamo Calo^3^, Shu Ye^1^, Jonathan P. Thompson^1^ and David G. Lambert^1^

^1^Department of Cardiovascular Sciences, Anaesthesia, Critical Care and Pain Management, University of Leicester, Hodgkin Building, Leicester, LE1 9HN. UK.

^2^Department of Chemical, Pharmaceutical and Agricultural Sciences, University of Ferrara, 44121 Ferrara, Italy.

^3^Department of Pharmaceutical and Pharmacological Sciences, University of Padova, 35131 Padova, Italy.

**SUPPORTING INFORMATION; SUPPLEMENT.**

**FIGURE-2**

**Methods: Immunofluoresence**

PN5 (HUVEC), human umbilical artery smooth muscle cells (HUASMC) and the fibroblast cell line MRC5 were seeded at 6000 cells/well into 0.2% gelatin-coated CellCarrier Ultra plates (Perkin Elmer) and allowed to adhere overnight. The next day, the cells were washed with PBS and fixed with 4% paraformaldehyde in PBS for 10 minutes. After fixation, the wells were washed once with PBS and the fixation quenched using 0.05M ammonium chloride in PBS. The wells were washed with PBS again and permeabilized with 0.2% Triton X-100 in PBS for 15 minutes. Following permeabilization, 1% bovine serum albumin (BSA, in PBS) was added and blocking was performed for 1 hour at room temperature. Next, primary antibodies were added for the endothelial marker vWF (ab194405, Abcam), the smooth muscle marker α-SMA (ab7817, Abcam) or the fibroblast marker TE-7 (CBL271, Sigma-Aldrich) in blocking buffer (1% BSA in PBS) in dilutions of 1:200, 1:200 and 1:100, respectively. The primary antibodies were incubated for 1 hour at room temperature, followed by 3 washes with blocking buffer for 10 minutes per wash. Next, the secondary antibody (goat anti-mouse IgG H&L Alexa Fluor 488, Abcam ab150113) was added to the wells at a dilution of 1:500 in blocking buffer for 1 hour at room temperature. After secondary antibody incubation, the wells were washed 3 times (10 minutes each) with blocking buffer before incubation with 0.4µM Hoechst 33342 for 1 hour to stain the nuclei blue. The wells were washed once with PBS and imaged using an Operetta CLS high-content imaging system (Perkin Elmer) using a 10x objective lens.

**Results**

**2. HUVEC-PN5 line is endothelium and not muscle.**


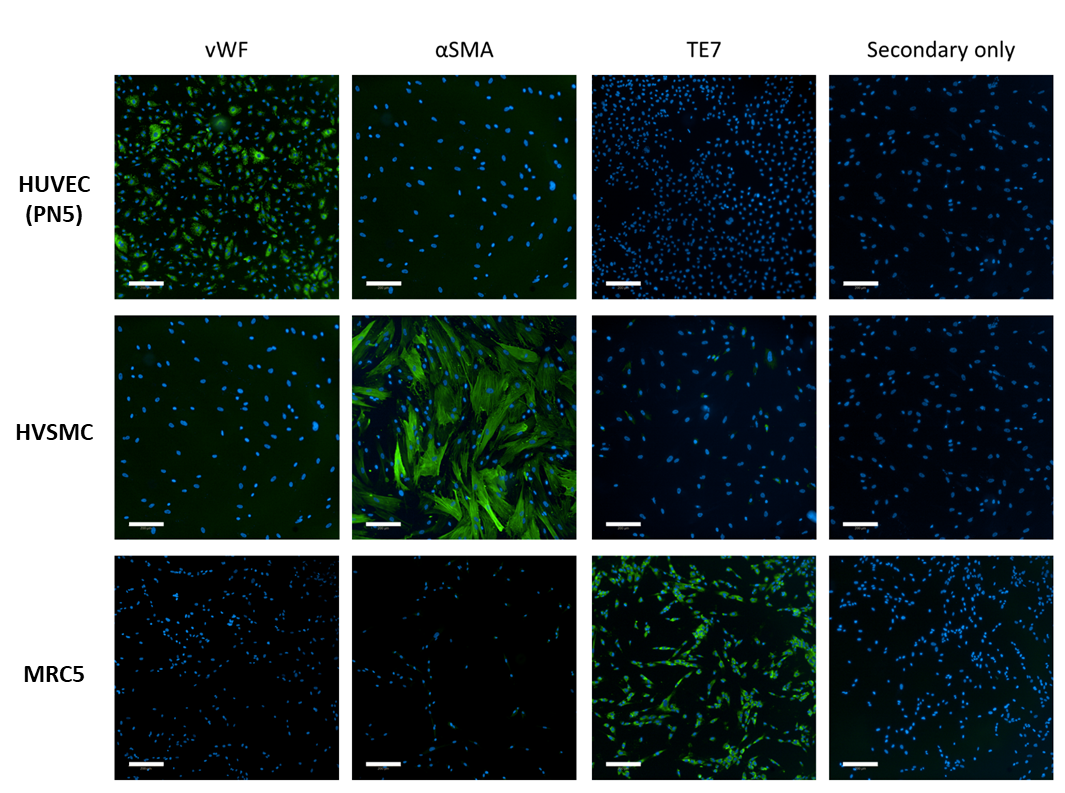


**Supplement Figure-2**: Immunofluorescence Analysis of HUVEC cell Line PN5. In order to confirm the cell type identity of line PN5, immunofluorescence for the endothelial marker von-Willebrand Factor (vWF), the smooth muscle cell marker α-smooth muscle actin (α-SMA) and the fibroblast marker TE-7 with smooth muscle cell (human umbilical cord artery cells; HVSMC) and fibroblast (MRC5) positive controls. A secondary antibody-only control was included for all cell types. Green fluorescence indicates positive staining for the indicated marker. Nuclei were counterstained with Hoechst 33342 (blue). Images were obtained using with an Operetta CLS high-content imaging system (Perkin Elmer) using a 10x objective lens. Scale bar = 200µm.
