# Supplementary material for: In vitro sepsis induces Nociceptin/Orphanin FQ receptor (NOP) expression in primary human vascular endothelial but not smooth muscle cells
Source: PLoS One. 2022 Sep 15;17(9):e0274080. doi: 10.1371/journal.pone.0274080 (PMC9477356; doi:10.1371/journal.pone.0274080)
Supplement: S1 Raw data — (PDF) [file pone.0274080.s003.pdf]

Western blot raw images

All images were obtained using BioRad ImageLab software as detailed in methods section of paper. Images are represented in the order they appear in the main texts. Molecular weights are included in these images (biotinylated ladder; #7727, Cell signalling). The red box indicates area chosen in figures.

Figure4 4A

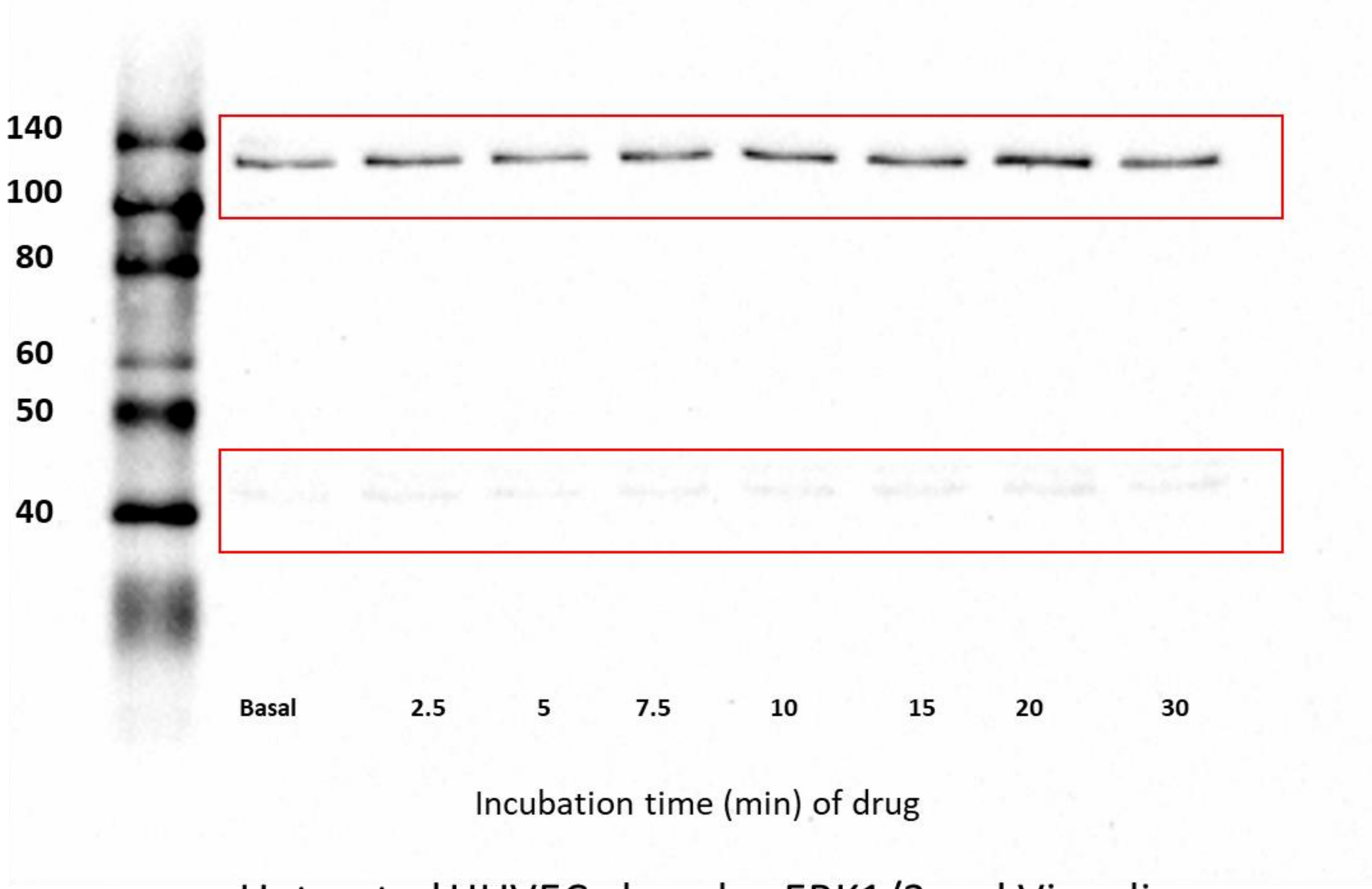

Untreated HUVEC phospho-ERK1/2 and Vinculin

Figure 4B

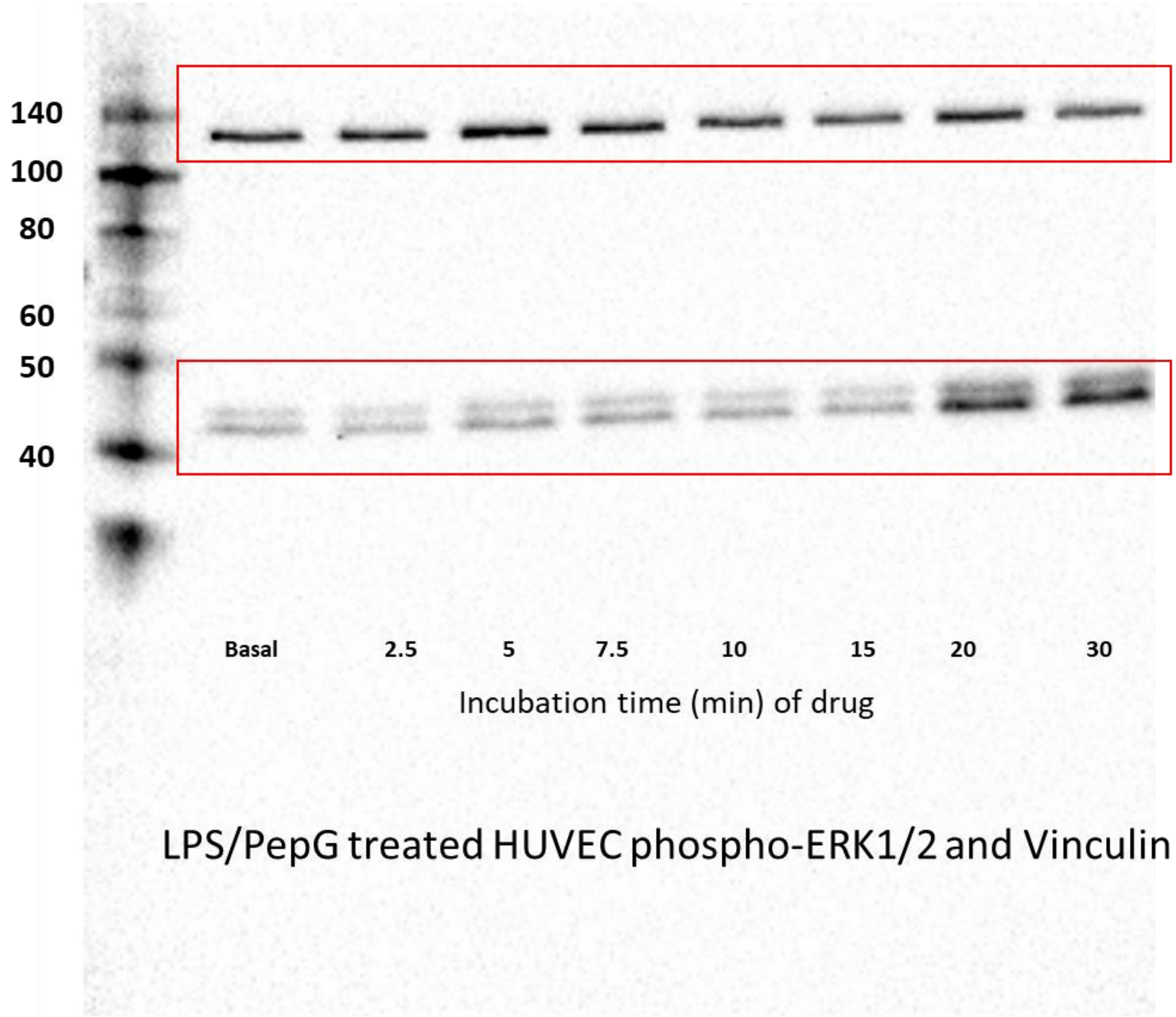
140
100
80
60
50
40
Basal
2.5
5
7.5
10
15
20
30
Incubation time (min) of drug
LPS/PepG treated HUVEC phospho-ERK1/2 and Vinculin

Figure 5:

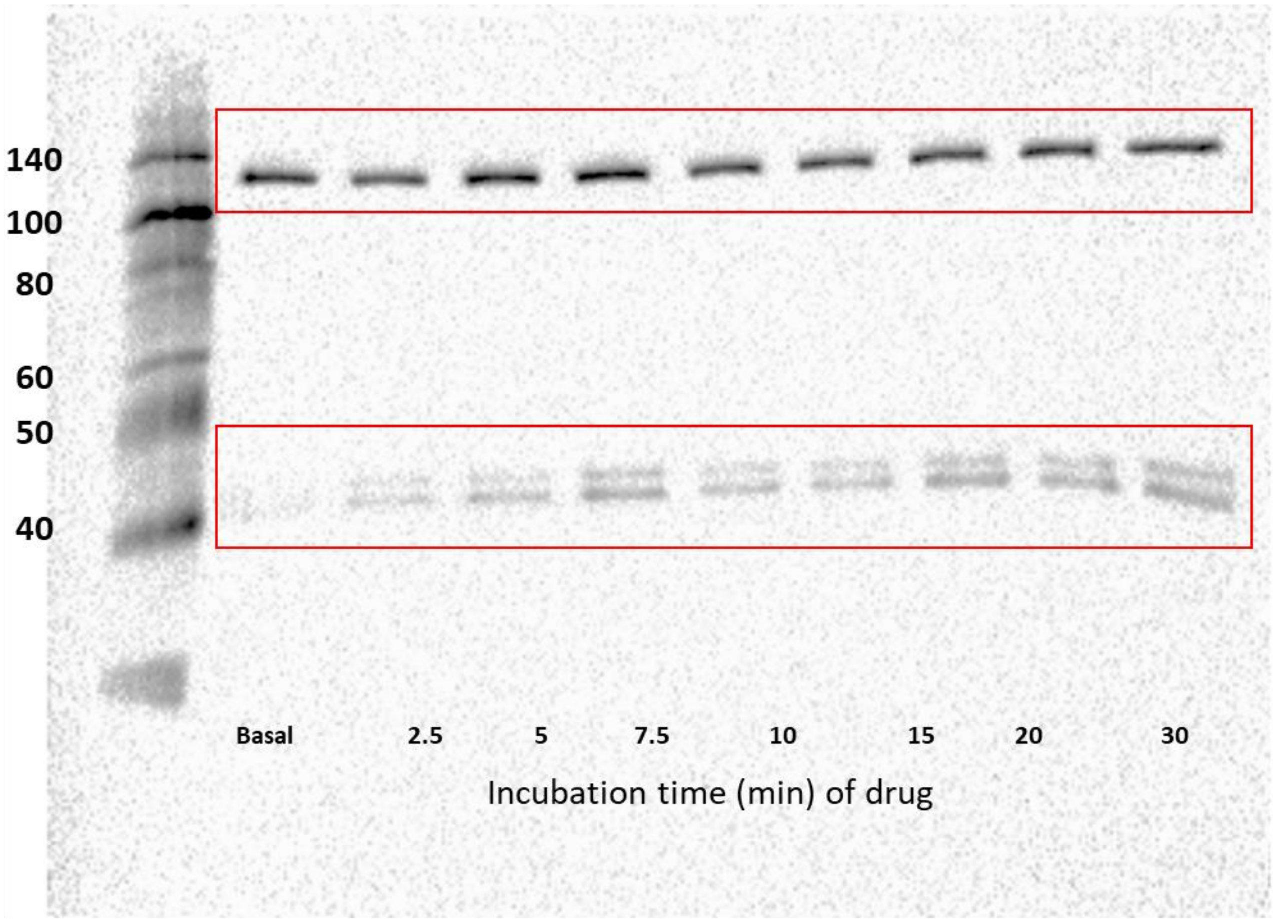

Untreated HUVEC PN5 phospho-ERK1/2 and Vinculin

Figure 7:

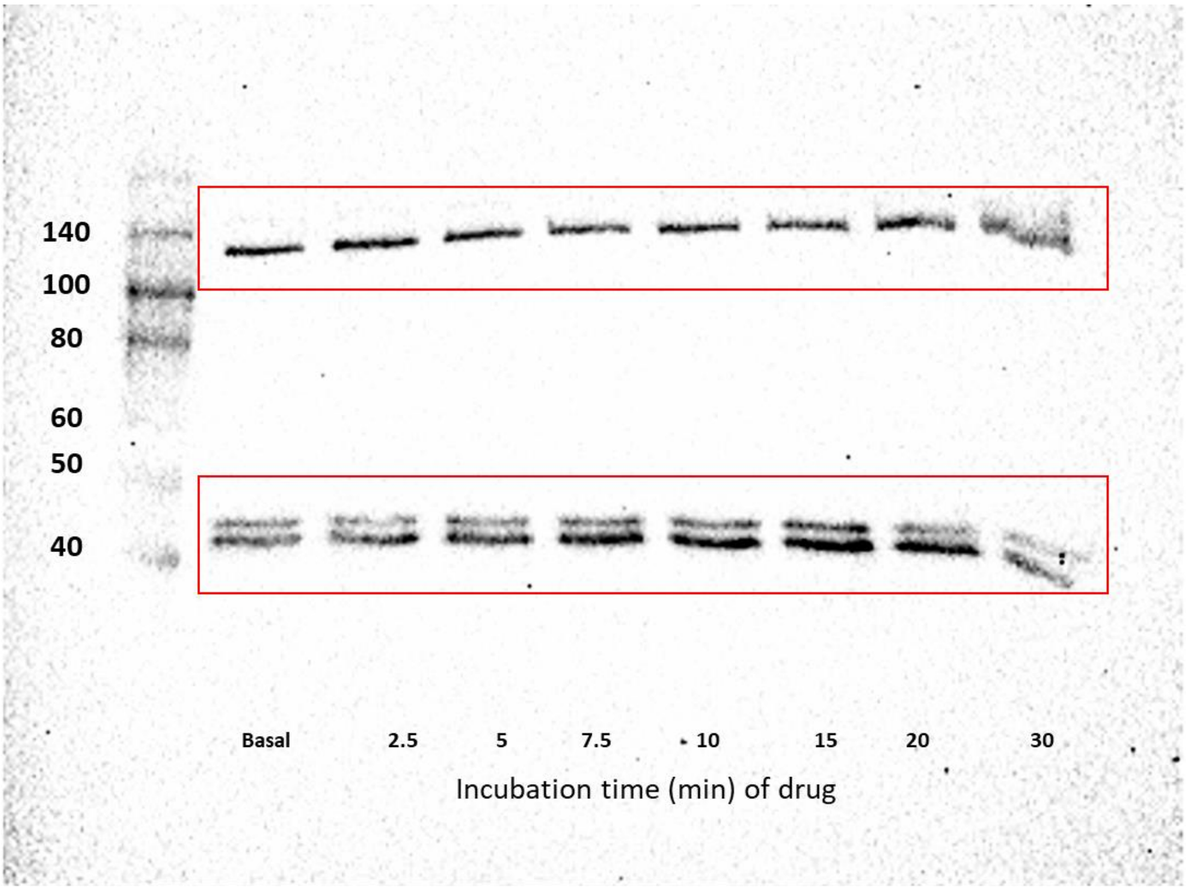

Untreated VSMC with phospho-ERK1/2 and Vinculin
